# Histological chorioamnionitis is associated with an increased risk of wheezing in preterm children less than 34 gestational weeks

**DOI:** 10.1186/s12887-021-02572-9

**Published:** 2021-03-01

**Authors:** Xiaoli Wang, Haiyuan Li, Qianqian Zhang, Qianwen Shen, Dan Zhu, Hong Li, Zheng Tang, Jiuru Zhao, Zhiwei Liu

**Affiliations:** 1 International Peace Maternity and Child Health Hospital, School of Medicine, Shanghai Jiao Tong University, 910# Hengshan Road, Shanghai, 200030 China; 2 International Peace Maternity and Child Health Hospital of China Welfare Institute, Shanghai, China; 3 Shanghai Key Laboratory of Embryo Original Disease, Shanghai, China

**Keywords:** Chorioamnionitis, Preterm birth, Outcome, Wheezing, Metabolomics

## Abstract

**Background:**

Chorioamnionitis is associated with various neonatal short- and long-term morbidities. The effect of chorioamnionitis on premature children’s outcomes remains controversial. The aim of this study is to investigate the relationship between histological chorioamnionitis (HCA) and physiological development, wheezing, and atopic diseases in preterm children.

**Methods:**

Singleton, preterm children (< 34 weeks), whose mother underwent pathological placental examinations, were retrospectively enrolled and the outcomes were assessed at 24–40 months during follow-up. Wheezing and atopic diseases including eczema, food allergies, and allergic rhinitis were screened by a questionnaire along with medical diagnosis. Anthropometric indexes and blood pressure were measured. Cognitive and behavioural developments were assessed by the Gesell Development and Diagnosis Scale. Blood IgE and routine examination were analyzed with venous blood and serum metabolomic profiling was assessed via liquid chromatography-mass spectrometry (LC-MS). A multivariate logistic regression model was used to estimate the association between HCA and the current outcomes.

**Results:**

Among the 115 enrolled children, 47 were exposed to HCA. The incidence of wheezing was significantly higher in children exposed to HCA, as 38.30% of children who were exposed to HCA and 16.18% of children who were not had been diagnosed with wheezing. After adjusting for related confounders in the multivariate logistic regression model, there remained a 2.72-fold increased risk of wheezing in children with HCA (adjusted odds ratio, aOR, 2.72; 95% confidence interval, 1.02–7.23). Moreover, 163 differential metabolites, such as butanoic acid, annotemoyin 1 and charine, were identified in the HCA exposed children’s serum. Enrichment analysis revealed that these compounds participated in diverse key metabolomic pathways relating to physical and neuro- developments, including glycerophospholipid, alpha-linolenic acid and choline metabolisms. There were no significant differences in atopic diseases, serum IgE, eosinophils’ level, anthropometric indexes, blood pressure, or cognitive or behavioural developments between the two groups.

**Conclusion:**

HCA exposure is associated with an increased risk of wheezing in preterm children less than 34 gestational weeks.

**Supplementary Information:**

The online version contains supplementary material available at 10.1186/s12887-021-02572-9.

## Background

Intrauterine events can have a lifelong impact on chronic diseases. Exposure to adverse stimuli can permanently change the structure and function of tissues and organs. Every year, nearly 14.9 million preterm babies are born, accounting for about 10.5% of total births [1]. Studies have revealed that preterm birth is associated with an increased risk of chronic diseases in adulthood and impairs neurodevelopment such as cognitive and motor impairments and hearing loss [2]. Chorioamnionitis is a common gestational complication which results in 40–70% of preterm deliveries. Diagnosis of histological chorioamnionitis (HCA) is mostly based on the infiltration of neutrophils and lymphocytes into the placenta and associated membranes. Many studies have associated chorioamnionitis with various short- and long-term adverse outcomes, such as intrauterine growth restriction, cerebral palsy, chronic lung disease, wheezing or asthma later in life [3, 4]. However, the outcomes are inconsistent across studies. For example, several studies found that chorioamnionitis increased the risk of long-term cognitive and behavioural impairments in preterm children [5, 6], while others did not [7].

Currently, substantial studies about the association between preterm birth and atopic diseases and long-term physical development have been published [8, 9]. However, studies about the effects of chorioamnionitis on wheezing and atopic diseases among preterm children less than 34 gestational weeks remain limited. There is also a lack of hematological indicators in previous studies. Furthermore, systemic measurements of the physiological developments of preterm children less than 34 weeks who were exposed to chorioamnionitis at 2–3.5 years of age have not been reported.

Based on previous reports, we hypothesized that HCA exposure would be associated with an increased impairment of children’s developments and risk of atopic diseases. Thus, in the present study, we followed up the outcomes related to anthropometric indexes, neurodevelopment, wheezing, atopic diseases (eczema, food allergies and allergic rhinitis) and allergic indicators among preterm children less than 34 weeks who were exposed to HCA. The aim of the study was to investigate the relationship between HCA and physiological development, wheezing, and atopic diseases in preterm children.

## Methods

### Study design

**Fig. 1:**
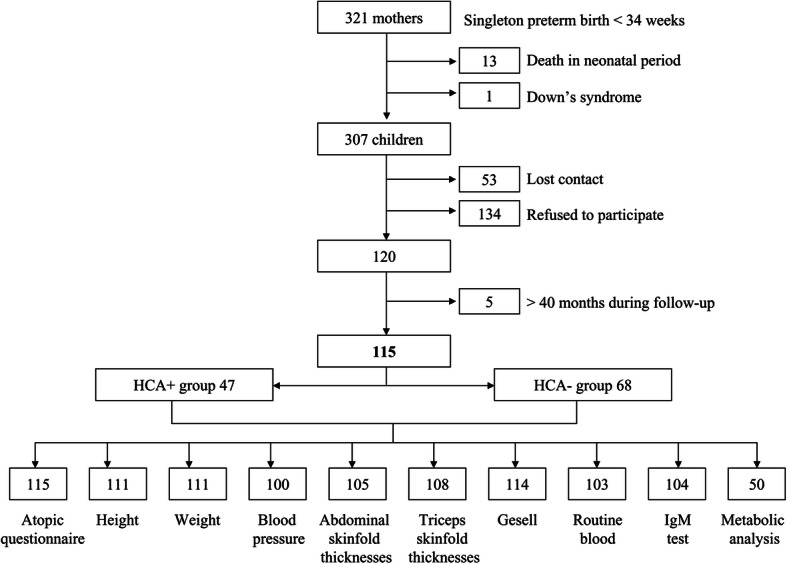
Flow chart of participants in the study

All women who delivered singleton preterm children (< 34 weeks) between June 2015 and August 2017 at our hospital and underwent pathological placental examinations were retrospectively enrolled. The follow-up of their children was conducted at 24–40 months postpartum. The exclusion criteria were: (1) children with chromosomal abnormalities, (2) children death in neonatal period. Totally, there were 307 eligible children and 115 children were followed up in the current study. The flow chart of participants is shown in Fig. 1.

Ethics approval was obtained from the ethics committees of our institute [approval number (GKLW) 2016–21]. Informed consents had been obtained from the parents of all subjects in accordance with the Declaration of Helsinki. All data were available upon reasonable requests.

### Data collection

#### Wheezing and atopic diseases

Data on wheezing and atopic diseases including eczema, food allergies, and allergic rhinitis were collected using a revised version of the third National Epidemiological Survey questionnaire on asthma in children [10]. Wheezing was determined based on medical diagnosis and the need for treatment with inhaled glucocorticoids and/or bronchodilators. Atopic diseases were diagnosed by physicians.

#### Neurodevelopment

Neurodevelopment was assessed using the revised version of the Gesell Development and Diagnosis Scale. This scale consists of five domains, including gross motor, fine motor, adaptability, language, and personal-social behaviour domains. Final results were represented by developmental quotient (DQ). A DQ < 85 in any domain was considered to be abnormally low [11]. Given that the applicable range of age for this scale is 0–42 months, children were limited to less than 40 months of age to ensure accuracy.

#### Anthropometric measurement

Height and weight were measured using a standard procedure. Physical retardation was diagnosed if either height or weight scored less than the 10th percentile for peers of the same age and sex according to the reference (published by Shanghai Center for Women and Children’s Health). Triceps and abdominal skinfold thicknesses were measured using the guidelines recommended by the World Health Organization.

#### Blood pressure

Children’s blood pressure was measured in the left upper arm brachial artery using a medical electronic sphygmomanometer (A&D Medical, USA).

#### Others

Data on the postnatal living environments and lifestyles of preterm children were collected via parental questionnaire. Clinical information about placental pathology and perinatal, maternal, birth, and neonatal conditions were extracted from the medical record system. Bronchopulmonary dysplasia (BPD) was defined as an abnormal chest x-ray and oxygen therapy that was still needed at 28 days after birth or at 36 weeks of corrected gestational age. Mechanical ventilation referred to invasive ventilation but did not include nasal continuous positive airway pressure or simple mask oxygen inhalation. Intrauterine growth restriction (IUGR) was defined as an estimated fetal weight of less than 10th percentile based on ultrasound and Doppler assessments. Patent dutus arteriosus (PDA) was diagnosed based on echocardiographic assessment or clinical symptoms (murmur, widened pulse pressure, active precordium). Gestational age (GA) was calculated according to the last menstrual period and early (< 20 weeks) ultrasound findings.

### Blood examination

To avoid variation due to circadian rhythms, blood was drawn in the morning between 8:00 and 11:00 after overnight fasting. Totally, 5 ml venous blood was collected. Blood samples were transferred into SST tubes and gently inverted twice, then rested at room temperature for 30 min. Serum was collected by centrifuging at 2000×g for 15 min at 4 °C. For children who were not willing to draw venous blood, routine peripheral blood test was carried out with fingertip blood.

IgE was measured by an enzyme-linked immunosorbent assay (ELISA, URANUS AE 65, Aikang Medical, China), which contained total IgE (tIgE) and 20 antigen-specific IgE (sIgE). sIgE included IgE against *Artemisia argyi*, *Dermatophagoides pteronyssinus*, *Alternaria*, *Dermatophagoides culinae*, common dolphin, house dust, willow, dog skin, cat skin, wheat flour, peanut, soybean, egg, milk, beef, mutton, crab, shrimp, codfish, and cockroach. tIgE exceeding 60 IU/ml or any sIgE exceeding 0.35 IU/ml was considered to be positive. An eosinophils’ ratio exceeding 5% was considered to be allergic.

For metabolomic analysis, 120 μl serum sample stored at − 80 °C from 25 children per group were thawed at 4 °C respectively and 10 μl each of 2-chloro-1-phenylalanin (0.3 mg/ml) and 17:0 Lyso PC (0.01 mg/ml) dissolved in methanol were added. The mix was vortexed for 10 s. Subsequently, 360 μl of ice-cold mixture of methanol and acetonitrile (2/1, v/v) was added. Samples were then vortexed for 1 min, ultrasonicated at 25 °C for 10 min, stored at − 20 °C for 30 min and centrifuged at 10000×g for 10 min at 4 °C. The supernatant (400 μl) was dried in a freeze concentration dryer, and 200 μl of a methanol and water mixture (1/4, v/v) was added. Samples were vortexed for 30 s, placed at 4 °C for 2 min, and centrifuged at 10000×g for 10 min at 4 °C. Supernatants were filtered though 0.22 μm microfilters before proceeding to LC-MS analysis (Shanghai Luming Biological Technology, China) with the ACQUITY UHPLC (Waters Corporation, USA) and Triple TOF 5600 (AB SCIEX, USA) systems.

Progqenesis QI software (Waters Corporation, USA) was used to identify metabolites in raw LC-MS data. Principal components analysis (PCA) and (orthogonal) partial least squares-discriminant analysis (PLS-DA, OPLS-DA) were used to visualize the metabolic alteration among groups with the R ropls package. Variable importance in the projection (VIP) was used to rank the overall contribution of each variable to the OPLS-DA model. Metabolites with adjusted *P* <  0.05 and VIP > 1 were considered to be differential. The altered pathways were enriched in the Kyoto Encyclopedia of Genes and Genomes (KEGG) database (http://www.genome.jp/kegg/) based on identified differential metabolites.

### Placental histopathological examination

Fresh placentas were fixed in 4% paraformaldehyde and embedded with paraffin. Seven samples were taken for microscopic assessment, including three blocks of the placental disk, two cross-sections of the cord (one near the fetus and the other placental insertion), and two membrane rolls. Placental inflammation was assessed by three independent pathologists. Chorioamnionitis was defined as polymorphonuclear leucocytes infiltrating the chorion, amnion, decidua, and chorionic plate with or without the involvement of the umbilical cord, per Redline’s standards [12]. The few cases of inconsistent diagnoses were confirmed by the consultations of pathologists.

### Statistics

Continuous data are presented as means ± standard deviations (SD). Categorical data are presented as frequencies and percentages. Student’s *t* or Mann-Whitney *U* tests were used to compare continuous variables and chi-squared or Fisher’s exact tests were used to compare categorical variables. After the descriptive univariate analysis, a multivariate logistic regression analysis approach was used to assess the association between chorioamnionitis and current outcomes with SPSS 24.0 software (IBM, USA). The confounders included maternal age, children’s current age, gender, type of delivery and use of antibiotics in the first year of life. The covariates were priori selected based on historical reports [13–15]. A causal model was firstly built and the confounders were tested for each model outcome. Covariates were selected or excluded based on the change in effect estimate of the variable of interest and the reduction of residual variance of the model. *P* <  0.05 (two-sided) indicated statistical significance.

## Results

In this study, a total of 307 eligible mother-children pairs were screened out, of which 115 were successfully enrolled (Fig. 1). Comparison of the maternal and children’s baseline characteristics revealed that there was nearly no significant difference between the recruited and study populations, except the maternal education level (Table S1). Among the 115 children, 47 (40.87%) were in the HCA exposed group and 68 in the unexposed group (Table 1). The mean gestational age was 31.66 ± 1.70 weeks. There were no significant differences between the two groups in maternal age, children’s birth weight, gender, and first degree relatives’ allergic history [16, 17]. Variables that were significantly associated with HCA included a smaller gestation age, preterm premature rupture of the membranes (PPROM), vaginal delivery and the use of antibiotics in the first year of life (Table 1).

**Table 1:** Maternal and children’s baseline characteristics

	HCA (*N* = 47)	No HCA (*N* = 68)	*P* value
Maternal demographic			
Maternal age, y	32.04 ± 3.75	32.03 ± 3.89	0.99
Maternal education, ≥ college	32 (68.08)^a^	50 (73.53)	0.53
Children demographic			
Gestational weeks	31.16 ± 1.93	32.00 ± 1.44	0.02^*^
Birth weight, g	1714.57 ± 425.12	1774.63 ± 385.29	0.43
Male	30 (63.83)	41 (60.29)	0.70
Pregnancy history			
Parity, nulliparous	32 (68.09)	44 (64.70)	0.71
History of abortion, ≥ 1	23 (48.94)	33 (48.53)	0.97
Pregnancy comorbidities			
Pre-eclampsia	4 (8.51)	15 (22.06)	0.05
GDM	10 (21.28)	10 (14.71)	0.36
Pregnancy medications			
Antenatal steroids	42 (89.36)	63 (92.65)	0.78
Antenatal antibiotic	38 (80.85)	60 (88.24)	0.27
PPROM	33 (70.21)	21 (30.88)	< 0.001^***^
Neonatal birth information			
Mode of delivery, vaginal	29 (61.70)	21 (30.88)	0.001^***^
Fetal distress	9 (19.15)	20 (29.41)	0.21
5 min Apgar score ≤ 7	4 (8.51)	3 (4.41)	0.61
BPD	2 (4.26)	2 (2.94)	1.00
Mechanical ventilation	7 (14.89)	8 (11.76)	0.62
Surfactant administered	10 (21.28)	17 (25.00)	0.64
PDA	7 (14.89)	7 (10.29)	0.46
Maternal allergic history^b^	10 (21.28)	17 (25.00)	0.64
First degree relatives’ allergic history	25 (55.32)	35 (52.94)	0.43
Use of antibiotics in the first year	44 (93.62)	54 (79.41)	0.03^*^
Pneumonia	15 (31.91)	17 (25.00)	0.42
Breast-feeding	18 (38.30)	18 (26.47)	0.18

^a^ Numbers in the brackets indicated the percentages

^b^ Maternal allergic history included eczema, allergic rhinitis, and asthma

^*^ and ^***^, *P* < 0.05 and *P* < 0.001

During the follow-up (ages 2–3.5 years), outcomes of physical development, blood pressure, and neurodevelopment were similar among the children exposed to HCA and those who were not (Table 2). However, preterm children exposed to HCA were significantly more prone to wheezing than control children (38.30% vs 16.18%; *P* < 0.05). After adjusting for maternal age, children’s current age, gender, type of delivery and use of antibiotics in the first year of life, multivariate logistic regression analysis revealed that preterm children who were exposed to HCA had a 2.72-fold increased risk of wheezing (aOR, 2.720; 95% confidence interval, CI, 1.02–7.23). Furthermore, neither univariate nor multivariate analyses revealed an association between HCA and eczema, food allergies, or allergic rhinitis (Table 3).

**Table 2:** Follow-up indexes and statistical results

	N (HCA/no HCA)^a^	HCA (*n* = 47)	No HCA (*n* = 68)	*P* value
Age, m	47/68	29.50 ± 4.73	30.22 ± 4.67	0.42
Anthropometic parameters				
Height, cm	45/66	90.20 ± 4.36	90.65 ± 4.70	0.61
Height ≥ P10^b^		30 (66.67) ^c^	46 (69.70)	0.74
Weight, kg	45/66	13.13 ± 1.74	12.99 ± 2.18	0.73
Weight ≥ P10		34 (75.56)	46 (69.70)	0.50
Tricep skin fold thickness	45/63	7.80 ± 1.90	8.00 ± 2.40	0.64
Abdomen skin fold thickness	43/62	6.40 ± 1.68	6.32 ± 2.30	0.86
BP	41/59			
SBP		96.56 ± 9.89	97.46 ± 9.54	0.65
DBP		59.95 ± 8.90	61.66 ± 9.24	0.36
Gesell	47/67			
Gross motor domain		101.91 ± 19.99	103.43 ± 13.72	0.65
DQ<85		4 (8.51)	5 (7.46)	1.00
Fine motor domain		88.55 ± 11.80	88.31 ± 14.15	0.92
DQ<85		16 (34.04)	30 (44.78)	0.25
Adaptive domain		103.45 ± 13.36	103.07 ± 13.73	0.89
DQ<85		4 (8.51)	5 (7.46)	1.00
Language domain		100.09 ± 19.48	100.49 ± 20.31	0.91
DQ<85		10 (21.28)	14 (20.90)	0.96
Personal-social domain		94.30 ± 11.30	98.90 ± 15.12	0.07
DQ<85		7 (14.89)	11 (16.42)	0.83
Wheezing	47/68	18 (38.30)	11 (16.18)	0.007^*^
Atopic diseases	47/68			
Eczema		20 (42.55)	28 (41.18)	0.88
Food allergy		17 (36.17)	21 (30.88)	0.55
Allergic rhinitis		10 (21.28)	11 (16.18)	0.49
Blood examination				
Eosinophils (10^9^/L)	44/59	0.33 ± 0.29	0.28 ± 0.20	0.29
EOS positive %		8 (18.18)	12 (20.34)	0.78
tIgE	43/61	171.31 ± 267.78	124.57 ± 203.66	0.52
tIgE positive		23 (53.49)	26 (42.62)	0.27
sIgE positive		32 (74.42)	44 (72.13)	0.80
Environmental exposure				
Passive smoking	47/68	12 (25.53)	19 (27.94)	0.78
Interior decoration	47/68	9 (19.15)	9 (13.24)	0.39
Carpet	47/68	2 (4.26)	2 (2.94)	1.00
Flower or grass	47/68	19 (40.43)	37 (54.41)	0.14
Mold/mildew in home	47/68	15 (31.91)	17 (25.00)	0.42

^a^ Numbers may not sum to total due to missing data

^b^ P10: 10th percentile of the same age and sex; BP: blood pressure; SBP: systolic BP; DBP: diastolic BP; EOS positive: eosinophils’ ratio exceeding 5%; tIgE: total IgE; tIgE positive: tIgE > 60.00 IU/ml; sIgE: antigen-specific IgE; sIgE positive: sIgE > 0.35 IU/ml

^c^ Numbers in the brackets indicated the percentages

^*^ *P* < 0.05

**Table 3:** Multiple logistic regression analyses on the association between HCA and wheezing or atopic diseases^a^

	N	Crude OR	95% CI	Adjusted OR	95% CI
Wheezing	29	3.22	(1.34, 7.70)	2.72	(1.02, 7.23)
Eczema	48	1.06	(0.50, 2.25)	0.93	(0.40, 2.21)
Food allergy	38	1.27	(0.58, 2.79)	1.09	(0.46, 2.58)
Allergic rhinitis	21	1.40	(0.54, 3.62)	1.39	(0.47, 4.09)

^a^ Co-variables adjusted in the model: maternal age, children’s current age, gender, type of delivery and use of antibiotics in the first year of life. Children’s age, maternal age and gestational age were incorporated in continuous variables. All analyses used the same model

**Fig. 2:**
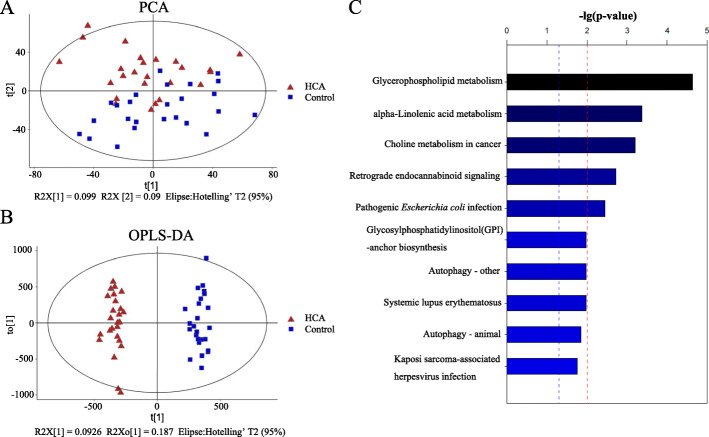
Serum metabolomic profile was changed in preterm children exposed to histological chorioamnionitis. **a** PCA analysis of the serum metabolomic profiles. t [1] and t [2] indicated the first and second principal components; R2X indicated the cumulative interpretation rate; Hotelling’s T2 region defined the 95% confidence interval of the model. **b** OPLS-DA analysis of the metabolomic profiles. **c** KEGG classification of the differentially identified metabolites

To further investigate the influence of HCA on preterm children, serum metabolomic profiling was conducted, which is a powerful tool for assessing metabolic alterations under a given set of physiological conditions. In total, 163 differential metabolites were identified, including 105 upregulated ones and 58 downregulated ones in preterm children who were exposed to HCA compared to those who were not (Table S2). PCA analysis revealed a clear separation between the HCA and control groups (Fig. 2a). To minimize the influence of intergroup variability and to further improve group separation, PLS-DA and OPLS-DA analyses were conducted. Similarly, the HCA group was clearly separated from the control group (Fig. 2b). In the OPLS-DA analysis, the R^2^ value indicated how well the data were mathematically reproduced, which ranged between 0 and 1 with 1 indicating the best model. The Q^2^ value indicated the percent variation of the response predicted by the model [18]. The results revealed an R^2^ of 0.906 and a Q^2^ of − 0.497, indicating that the model was stable and perfectly fit. The top differentially identified metabolites included 3-hydroxy-2-methyl-[R-(R,S)]G-butanoic acid, annotemoyin 1,and charine (Table 4). KEGG analysis of the differential metabolites revealed an enrichment of several key metabolic pathways, including the glycerophospholipid, alpha-linolenic acid and choline metabolisms (Fig. 2c). Specially, we found that the pathway of pathogenic *Escherichia coli* infection was involved in the metabolic changes associated with HCA (Fig. 2c), indicating a continuous influence of the intrauterine exposure since gram-negative bacterial infection is the most common cause of HCA.

**Table 4:** Top 20 differential metabolites in serum of preterm children exposed to histological chorioamnionitis^a^

Metabolites	Fold change	VIP^b^	Adjusted *P* value
Annotemoyin 1	3.30	1.80	2.33E-4
Charine	3.08	1.58	4.36E-4
PA(18:2(9Z,12Z)/0:0)	3.08	1.23	2.30E-4
PE-Cer(d14:1(4E)/21:0)	3.08	1.33	1.03E-2
Oxamniquine	2.66	1.26	8.22E-3
31-hydroxy-32,35-anhydrobacteriohopanetetrol	2.62	1.90	5.30E-4
N-Stearoyl tyrosine	2.42	1.36	2.91E-3
LysoPE(0:0/18:3(6Z,9Z,12Z))	2.30	1.67	1.84E-2
32,35-anhydrobacteriohopaneterol	2.13	3.09	6.73E-4
Palmitoyl glucuronide	2.09	1.50	2.96E-3
Ascorbyl stearate	2.08	2.02	2.00E-4
2,4-Diphenyl-1-butene	2.06	1.81	3.39E-4
Cohibin A	2.03	3.05	6.43E-5
5beta-Cholestane-3alpha,7alpha,24-triol	2.03	2.00	6.08E-5
Methyl jasmonate	2.03	1.26	3.54E-2
23-methyl-5Z,9Z-tetracosadienoic acid	2.01	1.89	3.07E-4
25-Acetylvulgaroside	1.99	2.65	3.91E-3
Ambrettolic acid	1.96	4.13	2.22E-2
24-ethyl-5alpha-cholest-25-en-3alpha,12alpha,16alpha-triol	1.93	1.55	1.46E-3
3-Hydroxy-2-methyl-[R-(R,S)]-butanoic acid	0.34	3.58	5.55E-15

^a^ Metabolites with VIP > 1.20 and adjusted *P* < 0.05 were shown

^b^ *VIP* Variable importance in the projection

## Discussion

In chorioamnionitis, local levels of cytokines such as IL-1, IL-6 and TNF-α are increased. This disorder further induces multiple organ injuries [19]. Moreover, elevated proinflammatory mediators and inflammatory cytokines can induce life-long epigenetic modifications, which alter the development and function of cells. In this study, the follow-up demonstrated that HCA was associated with an increased risk of wheezing in preterm children less than 34 gestational weeks. Additionally, there was a significant alteration in serum metabolites. Our results contributed a further stone for the theory of intrauterine original disease.

Since the first report on the association between chorioamnionitis and early lung inflammation by *Watterberg* et al [20], the prenatal influence on lung development has been extensively studied. A cohort study of 1096 children found a joint effect of chorioamnionitis and prematurity on early children’s recurrent wheezing [21]. Another study of preterm children less than 32 gestational weeks also reported that fetal inflammatory response syndrome increased the risk of early childhood wheezing [22]. In this study, we found a 2.72-fold increased risk of wheezing in preterm children less than 34 weeks with HCA. However, in full-term children, reports indicated there was no such an association [23]. Studies in asthma showed that chorioamnionitis increased the risk of asthma, but the effect would diminish with increased gestational age [15]. Similar characteristics may also exist for the relationship between chorioamnionitis and early childhood wheezing, though further study is required.

Despite the increased risk of wheezing in preterm children exposed to chorioamnionitis, we did not observe significant differences in serum total or specific IgE in this study. There are at least two possible reasons for this: (1) the large variation in the levels of serum IgE among population; (2) the sampling time, since the children with wheezing in both groups were not all in an ongoing status. Nonetheless, we found children with chorioamnionitis were more prone to have a higher level of plasma eosinophils. Besides, metabolomic profiling revealed the alteration of serum metabolites from hypoxia and immune responses, and the lipid metabolism in HCA exposed children (Fig. 2c), which were fingerprints of asthma [24]. Reports indicated there was a linear correlation between early childhood wheezing and asthma among preterm children exposed to chorioamnionitis [25]. Thus, these results reinforce our finding that an increased risk of wheezing occurs in preterm children exposed to HCA.

Increased risk of IUGR and poor early postnatal growth in children exposed to chorioamnionitis has been widely reported. However, limited literatures have been published about the effect of chorioamnionitis on long-term physical development in premature children, and outcomes remain controversial [26, 27]. In this follow-up, we found no significant difference in height, weight, skinfold thickness or blood pressure between the preterm children exposed to HCA or not at the age of 2–3.5 years. Infancy and early childhood are critical periods for physical development. Our findings indicated that there might be no influence of chorioamnionitis on children’s physical development. However, the long-term physical disorders still require further investigations [28].

Chorioamnionitis may induce brain injury in preterm newborns and be linked to later adverse neurodevelopment. Thus, in the present study, children’s neurodevelopment was also evaluated. However, we found that there was no association between HCA and poor neurodevelopment in early childhood. This finding is consistent with recent reports [7, 29], which revealed an adverse association between chorioamnionitis and brain injury or developmental disability [6]. This discrepancy may be due to several factors including: (1) maternal education, which is associated with cognitive and behaviour developments in children (only 4.32% of mothers in our study did not receive an education at or above the high school level), and (2) antenatal steroid administration (91.26% of mothers in the present study received antenatal steroids), which protects the fetal brain from injury and decreases the risk of adverse neonatal outcomes.

Metabolomic profiling can provide insight into active physiological status, which has been proved to be useful in diagnosis of disease, monitoring prognosis and evaluation of therapeutic strategies. For example, formate, hippurate and methanol were proved to be common biomarkers of asthma [30]. In this study, we observed a clear separation of the serum metabolites between the HCA and control groups. Many altered metabolites or altered metabolic pathways have been reported to play key roles in children’s growth and development. For example, linolenic acid metabolism was reported to be associated with neurodevelopment [31]. Levels of metabolites can change before the emergence of phenotype. The minimal impacts observed during this follow-up may not tell the full story. Noteworthy, metabolomic analysis provides the field with a new perspective on the mechanisms underlying chorioamnionitis impacts on long-term chronic diseases. Thus, prompt clinical intervention in HCA cases might reduce the prevalence of wheezing in offspring.

There were some potential limitations that need to be considered when interpreting our data. First, the sample size of present follow-up was relatively small. For example, while the aOR for wheezing was 2.72, the 95% CI stretched across 1.02 to 7.23. Meanwhile, inconsistent with previous studies [7, 23, 29], the other observed children’s outcomes of HCA exposure was modest in this study. Although there was nearly no difference between the recruited and study populations (Table S1), statistical power calculation revealed that the recruited number was lower than the required for the outcomes of low prevalence, such as DQ < 85 and physical retardation. A further limitation to this study was that postnatal nutrition data were not collected. Numerous studies have demonstrated the direct relationship of children’s physical development and nutrition [32]. The sodium salt intake is also strongly associated with blood pressure. Another limitation was the basis of socioeconomic status. More than 70% mother of the present study received education above college, which was much higher than the average of China. Reports have indicated socioeconomic status was strongly associated with children’s developments [14].

## Conclusions

In this study, we found that HCA was associated with an increased risk of wheezing among preterm children less than 34 weeks. Moreover, HCA induced a significant change of serum metabolomic profile in preterm children. However, HCA was not linked to atopic diseases, abnormal blood pressure, or physical or neuro- development.

## Supplementary Information

**Additional file 1: Table S1.** Maternal and children’s baseline characteristics of the recruited and study populations.

**Additional file 2: Table S2.** All differential metabolites identified in the serum of preterm children exposed to histological chorioamnionitis.

## Data Availability

The datasets supporting the conclusions of this article are included within the article and its additional files.

## Abbreviations

BP: Blood pressure
BPD: Bronchopulmonary dysplasia
CI: Confidence interval
DQ: Developmental quotient
ELISA: Enzyme-linked immunosorbent assay
GA: Gestational age
HCA: Histological chorioamnionitis
IUGR: Intrauterine growth restriction
KEGG: Kyoto Encyclopedia of Genes and Genomes
LC-MS: Liquid chromatography-mass spectrometry (LC-MS)
OPLS-DA: Orthogonal partial least squares-discriminant analysis
OR: Odds ratio
PCA: Principal components analysis
PDA: Patent dutus arteriosus
PLS-DA: Partial least squares-discriminant analysis
PPROM: Preterm premature rupture of the membranes
SD: Standard deviations
VIP: Variable importance in the projection
