# Supplementary material for: Histological chorioamnionitis is associated with an increased risk of wheezing in preterm children less than 34 gestational weeks
Source: BMC Pediatr. 2021 Mar 1;21:104. doi: 10.1186/s12887-021-02572-9 (PMC7919088; doi:10.1186/s12887-021-02572-9)
Supplement: Supplementary file 1 — Additional file 1: Table S1. Maternal and children’s baseline characteristics of the recruited and study populations. [file 12887_2021_2572_MOESM1_ESM.doc]

**Table S1. Maternal and children’s** baseline characteristics of the recruited and study populations

|  | Recruited (n = 115) | Loss follow-up (n = 206)^a^ | *P* value |
| --- | --- | --- | --- |
| Maternal demographic |  |  |  |
| Maternal age, y | 32.03 ± 3.56 | 31.54 ± 4.52 | 0.28 |
| Maternal education, ≥ college | 82 (71.30)^b^ | 120 (58.25) | 0.03* |
| Children demographic |  |  |  |
| Gestational weeks | 31.66 ± 1.58 | 31.41 ± 1.78 | 0.24 |
| Birth weight, g | 1750.11 ± 401.32 | 1696.04 ± 452.20 | 0.29 |
| Male | 71 (61.74) | 126 (61.17) | 0.92 |
| Pregnancy history |  |  |  |
| Parity, nulliparous | 76 (66.09) | 150 (72.82) | 0.21 |
| History of abortion, ≥ 1 | 56 (48.70) | 92 (44.66) | 0.49 |
| Pregnancy comorbidities |  |  |  |
| GDM^c^ | 20 (17.39) | 42 (20.39) | 0.51 |
| Pregnancy medications |  |  |  |
| Antenatal steroids | 105 (91.30) | 176 (85.44) | 0.13 |
| Antenatal antibiotic | 98 (85.22) | 171 (83.01) | 0.61 |
| PPROM | 54 (46.96) | 76 (36.89) | 0.08 |
| HCA | 47 (40.87) | 77 (37.38） | 0.54 |
| Neonatal birth information |  |  |  |
| Mode of delivery, vaginal | 50 (43.48) | 92 (44.66) | 0.84 |

^a^ Numbers may not sum to total due to missing data.

^b^ Numbers in the brackets indicated the percentages.

^c^ GDM, gestational diabetes mellitus; PPROM, preterm premature rupture of the membranes; HCA, histological chorioamnionitis.

^*^ *P* < 0.05.
